# A virtual reality craving study in tobacco addiction: The role of non-pharmacological support in tobacco detox therapy

**DOI:** 10.3389/fpsyt.2022.940100

**Published:** 2022-10-13

**Authors:** Lorenzo Zamboni, Simone Campagnari, Rosaria Giordano, Francesca Fusina, Silvia Carli, Alessio Congiu, Isabella Barbon, Silvia Melchiori, Rebecca Casari, Elisa Tedeschi, Roberta Vesentin, Giuseppe Verlato, Maurizio Valentino Infante, Fabio Lugoboni

**Affiliations:** ^1^Unit of Addiction Medicine, Department of Internal Medicine, G.B. Rossi Hospital, Verona, Italy; ^2^Department of Neuroscience, Biomedicine and Movement, University of Verona, Verona, Italy; ^3^Department of General Psychology, University of Padova, Padua, Italy; ^4^Padova Neuroscience Center, University of Padova, Padua, Italy; ^5^Diagnostics and Public Health-Unit of Epidemiology and Medical Statistics, University of Verona, Verona, Italy; ^6^Thoracic Surgery Department, University and Hospital Trust-Azienda Ospedaliera Universitaria Integrata, Verona, Italy

**Keywords:** tobacco, virtual reality, craving, nicotine, addiction

## Abstract

Nicotine addiction is a widespread, worldwide epidemic, causing six million deaths per year. A large variety of treatments for smoking cessation are currently available, including Cytisine, which is a promising drug due to its low cost and high safety levels. Notwithstanding the important amount of research on tobacco addiction treatments, smoking remains one of the most difficult substance use disorders to treat, probably also due to the fact that pharmacological treatment often overlooks other maintaining factors in this addiction, such as sensory impact and cue reactivity. To address this gap in both treatment protocols and scientific literature, we propose a study protocol in which we will compare the effects of combining Cytisine with Nirdosh, a herbal tobacco substitute, to Cytisine only in two groups of patients (C + N and C) who will also undergo exposure to four different virtual reality settings that will assess the importance of environmental cues. We will further assess mood and craving in the two samples, and include a control group taken from the general population. We expect the C + N group to report a more positive mood and a lower sensitivity to tobacco-related environmental cues.

## Introduction

The tobacco epidemic is a major threat to human health and economic development. With one billion smokers in the world and six million deaths per year (1), nicotine addiction is the most frequent substance use disorder (SUD) in the world. To date, several treatments for tobacco addiction (TA) are available, one of which is cytisine. Cytisine is considered to be the oldest medication for smoking cessation and has been used for this purpose in some Eastern/Central European and Central Asian countries for over 50 years (2, 3). Like Varenicline, it is a partial agonist of the nicotinic acetylchloline receptors (nAChRs) with a high affinity for the alpha-4 beta-2 nAChRs subtype, and its main action is to reduce withdrawal symptoms following smoking cessation (4, 5). Several sources points toward cytisine’s efficacy and effectiveness; it’s well tolerated when taken at the recommended dose, and adverse events reported in trials are typically non-serious and self-limiting gastrointestinal and sleep disturbances (6–8).

To date, we know that pharmacology therapy is not enough to counter smoking addiction. In several cases it is important to couple Cytisine detox therapy with other non-nicotinic treatments. Anti-smoking pharmacological medications address only the physical component of smoking (i.e., nicotine addiction), but they do not resolve smoking’s psychological components (cognitive, craving, social and behavioral including handling, holding and puffing a cigarette) (9, 10).

Craving, in particular, has been described as “the most fundamental and difficult problem for smokers who are trying to quit” (11). Several studies have been aimed at explicating the nature of tobacco craving and the factors that determine it. Craving is still conceptualized as a withdrawal symptom in some scientific literature (12), but this conceptualization doesn’t explain various phenomena related to smoking addiction, such as the fact that smokers can experience high levels of craving even when they are smoking freely and are clearly not in a state of withdrawal (13). Moreove1r, the same levels of craving can be higher after a short abstinence (14). More generally, several studies show that craving is largely determined by smoking-related cues (SRC) and expectations. SRC, such as the handling of cigarettes and exposure to smoking-related objects or contexts, can provoke powerful craving responses in smokers (15).

Cue reactivity (CR) is a hypersensitivity to stimuli and motivational situations (16). CR has a relevant role in addiction because it may increase craving and relapse risks, since subjects with a history of addiction are more sensitive to substance-correlated stimuli (17).

Cue reactivity is an evolutive phenotype, a biological characteristic of interaction with the environment. While a familiar space with many trigger stimuli could increase craving, an environment without trigger stimuli could on the contrary prevent relapse in tobacco addiction (18).

### Virtual reality

Virtual reality (VR) is a methodological approach that allows to recreate a realistic ecological representation of a tobacco craving-inducing situation. This approach has been used in several studies of cue reactivity (CR) in TA, especially for tobacco craving (19, 20).

Smoking is reinforced by a variety of sensory experiences (taste, smoke etc.) (21).

As of yet, none of the U.S. FDA-approved smoking cessation medications are specifically designed to address the “sensory impact” smokers report as desirable, satisfying, and reinforcing to their smoking behavior (22, 23). Sensory impact includes factors such as throat scratch, heat or coolness in the upper and lower airways, flavor (24, 25), and various sensations on the tongue, nose, throat, windpipe, and chest (22, 26). Sensory impact as a factor affecting cessation outcomes is critical to consider because, while outcomes improve with the use of approved cessation medications as compared to no treatment, relapse rates among treated smokers are still estimated to be as high as 50% 1 year post-cessation (27, 28).

Currently, there are several study related to the use of e-cigs for quitting and harm reduction in TA treatment (29, 30). To date, however, there are no studies about cytisine treatment and VR exposure in scientific literature.

### Cytisine

Cytisine is an alkaloid that is present in the *Cytisus laburnum*, a plant which is widespread in Central, Eastern and Southern Europe; this chemical has been used since the Sixties as a treatment for tobacco cessation.

As an active substance, cytisine is approved in Italy, but no pharmaceutical company produces it. It is, however, possible to find it in galenic form in virtually any pharmacy.

Cytisine is a partial agonist of the nicotinic receptors for acetylcholine, antagonizing both the nicotinic and the endogenous effects of acetylcholine (3, 5, 31, 32).

Many studies have shown that, in nicotine addiction, cytisine could, on one hand, only slightly increase the level of dopamine in the mesolimbic system (to about half the levels that nicotine induces). On the other hand, however, by limiting withdrawal symptoms cytisine could reduce the rapid spike in dopamine levels due to the rapid nicotine intake associated with a cigarette puff: indeed, cytisine is an excellent ligand of nicotinic receptors, presenting an affinity for the receptors α4β2 that is seven times higher than nicotine’s (33, 34).

Most studies on the pharmacokinetics of cytisine have been conducted in animals, with few human studies present in literature. The half-life of the drug is approximately 4.5 h and its elimination is mainly renal; a metabolization process does not take place, with 95% being eliminated through urine (35, 36).

Thanks to these characteristics, we can exclude any interactions with other drugs, as well as alterations of the pharmacokinetics in case of liver failure; on the other hand, there is a lack of studies on the pharmacokinetics of cytisine in patients with kidney failure.

Cytisine treatment is generally well tolerated. The most frequent adverse reactions are: changes in taste, dry mouth and throat, decreased appetite and, in rare cases, nausea. Headache and irritability have been observed in some patients on the first day of therapy. Among the side effects, no significant weight gain was found, but a significant increase in blood pressure values (just under 3 mmHg) was observed.

When taken at the recommended doses (1.5 to 9 mg/day for 25 days), cytisine is not associated with an increased risk of side effects compared to placebo, although gastrointestinal symptoms are more frequent (7, 37).

High doses can also cause dizziness and muscle weakness. All these effects pass quickly by reducing the dosage. Cytisine does not induce psychophysical alterations and therefore can also be taken by those who drive vehicles or operate machinery.

Since 2016, the Azienda Ospedaliera Universitari Integrata of Verona (AOUI) has been supplying 1.5 mg Cytisine capsules for the in-hospital treatment of patients with the aim of providing cost-effective support for the treatment of smoking, promoting therapeutic continuity even after discharge and reducing the risk of relapse.

The dosage of Cytisine used for this study is that by “(38),” visible in the table below. This protocol is based on the dosage recommended by the manufacturer, and starts with 1 tablet (1.5 mg) every 2 h (up to 6 tablets per day) on days 1 to 3, coupled with concurrent smoking reduction to avoid symptoms of nicotine overdose. The patient then continues with a dosage of up to 5 tablets per day (1 tablet every 2.5 h) from days 4 to 12. Smoking must be stopped on day 5. After that, the patient continues with 4 tablets per day (1 tablet every 3 h) from days 13–16, then proceeds with 3 tablets per day (1 tablet every 5 h) on days 17–20, followed by 1 to 2 tablets per day (1 tablet every 6–8 h) from the 21st to the 25th day, then the treatment is stopped. (39).

### Nirdosh

Nirdosh is a cigarette tobacco substitute (tobacco and nicotine free), registered to the Italian Health Ministry (registration code 1349698/R) produced by Herborea SRL (Company code 169252) and designed to help subjects during smoking cessation.

Nirdosh is a herbal mixture composed by basil, turmeric, licorice, cinnamon, cloves, tendu, sprague, and guggul.

We have chosen Nirdosh because it is simple to find, it is nicotine and tobacco free, and a registered medical device.

The primary outcome of this study protocol is to evaluate tobacco craving in 3 groups of participants; the secondary outcome is to evaluate the impact of Nirdosh in TA treatment.

Exclusion criteria:

• Pregnancy
• Unstable angina
• Pheochromocytoma
• Malignant hypertension
• COPD
• Epilepsy
• Psychosis
• Anxiety and depression severe
• Illicit drugs and alcohol dependence

Inclusion criteria:

• Tobacco addiction
• 18–65 years old
• Informed consent signature
• Prescription of cytisine according to the indications for use listed below

Dosage schedule for treatment with cytisine.

The following dosage schedule will be proposed ((38); Table 1).

**TABLE 1:** Graduated dosage schedule (38).

Days	N. cps/Die	Frequency of intake
1–3	6	1 cps every 2 h
4–12	5	1 cps every 2,5 h
13–16	4	1 cps every 3 h
17–20	3	1 cps every 4 h
21–25	2	1 cps every 6 h

cps, capsule.

Cytisine and Nirdosh will be provide free of charge to the study’s participants.

## Procedure

### Development and creation of virtual scenarios

**FIGURE 1:**
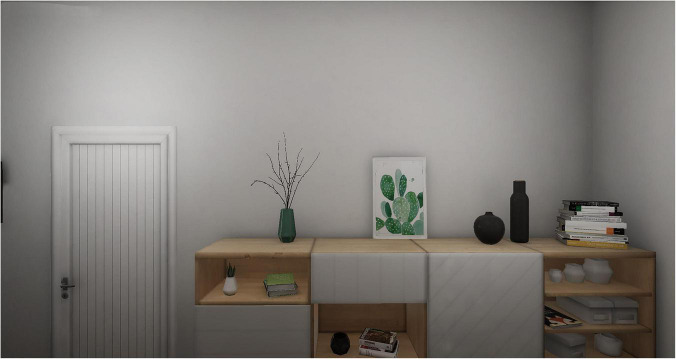
Neutral scenario.

**FIGURE 2:**
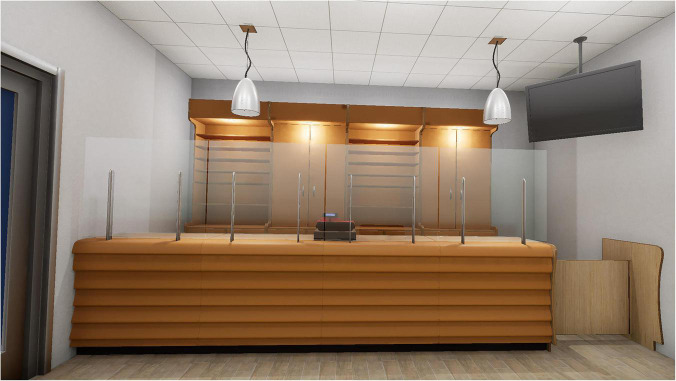
An empty tobacco store.

**FIGURE 3:**
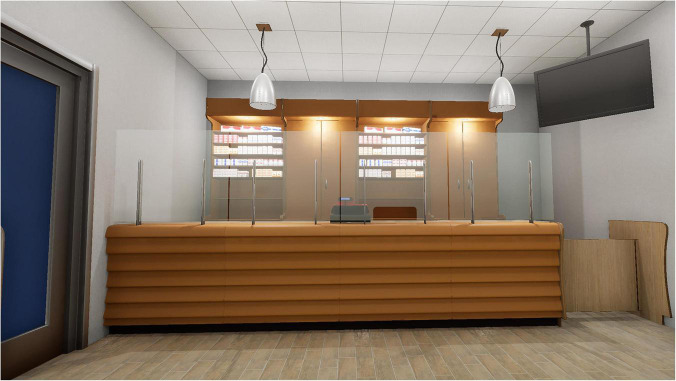
A tobacco store with cigarette packs.

We developed, created, and tested four virtual scenarios:

1. A cue-free tutorial scenario (VR tutorial), through which the subject can become familiar with the virtual reality instrumentation to learn how to move and explore each virtual environment.
2. A cue-free domestic entrance (neutral scenario, Figure 1).

3. An empty tobacco store (Figure 2).

4. A tobacco store with cigarette packs (Figure 3).

The subjects will be able to move within the virtual environments, but they will not be able to interact with the objects, except in the tutorial scenario. To reduce the possible insurgence of cybersickness [headaches, nausea, vomiting, dizziness; (40)], the subjects will be able to explore the virtual environments not only with the teleportation mode through the controllers, but also with real steps, which will be faithfully reproduced thanks to a virtual positional tracking.

The VR hardware supply is composed by: HTC Vive PRO Full Kit with wireless adapter; PC-Gaming Intel Core i7-9700K - GeForce RTX 2070 8GB - 16GB DDR4 - 480GB SSD - Windows 10 - WiFi; 49” or 55” TV monitor.

### Safety and hygiene measures

Due to the COVID-19 pandemic, we will guarantee safety and hygiene in the virtual setting through these procedures: wearing of surgical masks; hand cleaning with an alcohol-based hand sanitizer; usage of waterproof foam replacements on the HMD that will be sanitized easily; sanitization of all hardware devices after each use with alcohol-based products.

### Recruitment

The selection of the sample will be based on the inclusion criteria. A total of 78 subjects will be recruited in the Addiction Medicine Unit (AMU). They will be divided in three subgroups composed by 26 subject each. First we will recruit a general population group, to which participants will be assigned regardless of whether they smoke or not (GA group); then, two groups of smokers comprising patients that access the AMU’s antismoking center will be created by randomly assigning patients to a group which will follow a nicotine detoxification program with Cytisine plus Nyrdosh (C + N group), and a second group which will follow a Cytisine-only detoxification program (C group).

### Experimental procedures

During the first session, all subjects will sign the informed consent and will be interviewed for the collection of anamnestic and smoking history details.

**FIGURE 4:**
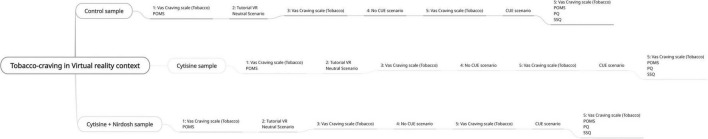
Study flow chart.

The GA group will be required to attend a one-hour single session, in which they will fill out the POMS and VAS will be exposed to the 4 virtual scenarios according to the following 8 virtual protocol steps (Figure 4):

1. Compilation of POMS and craving VAS.
2. Exposure to VR tutorial for 3 min.
3. Exposure to the neutral scenario for 3 min.
4. Compilation of craving VAS.
5. Exposure to the empty tobacco store for 3 min.
6. Compilation of craving VAS.
7. Exposure to the tobacco store with cigarette packs.
8. Compilation of POMS, craving VAS, Simulator Sickness Questionnaire (SSQ) and Presence questionnaire (PQ).

The GA group will follow a different procedure compared to C + N group and C group, but the virtual 8 steps will remain the same for all groups.

The C + N and C groups will be involved in the following steps. They will be required to attend four one-hour-and-a-half sessions structured as follows:

1. First psychological session: the psychologist will give the medical appointment to the patient at the end of the visit.
2. First medical appointment and exposure to the virtual protocol following the 8 steps above; in this step, the doctor will prescribe Cytisine and will explain how to take the drug (Table 1). Two packets of Nyrdosh will be given to the C + N group only.
3. Second medical visit and second exposure to the virtual protocol above, 7 days (± 2) after smoking cessation;
4. Third medical visit and third exposure to the virtual protocol above, 25 days (± 2) after ceasing smoking.

### Questionnaires

In this protocol we will use several self-report questionnaires:

● Psychological assessment:
  ○ Personal information: gender, age, marital status, drug status, alcohol use/abuse, medical information.
  ○ Tobacco anamnesis: cigarette (daily), years of tobacco addiction, psychiatric comorbidities, other substances used, health information and previous detoxification programs attended.
  ○ Nicotine dependence levels will be measured by the Fagerstrom test for nicotine dependence (FTND), which is a widely used test for assessing physical nicotine dependence (41).
  ○ Beck Depression Inventory II (BDI-II): a widely used self-report questionnaire which measures depressive symptomatology (42). The BDI-II is composed by 21 items rating on a 4-point Likert scale (0–3 points per item), with a higher score for more severe symptoms (43, 44).
  ○ Beck Anxiety Inventory (BAI): a self- report scale that was developed by Beck et al. (45). The BAI is one of the most used and easy to understand. The questionnaire comprises 21 items which assess the cognitive, emotional, and physical dimensions of anxiety. Each item is measured *via* a 4-point Likert scale that ranges from “strongly disagree (0)” to “strongly agree (3).” The total score ranges 0–63 points. The Cronbach’s α in the validation study was 0.93.

● Medical visit:
  ○ Profile of Mood State questionnaire (POMS): The POMS questionnaire comprises 65 items assessing the mood of the individual. A total mood disturbance (TMD) score is calculated by summing the totals for the five negative subscales, which are Tension, Depression, Fatigue, Confusion, and Anger, and then subtracting the positive subscale of Vigor. The POMS total score ranges from 0 to 60. High scores for tension, depression, anger, fatigue, confusion, and TMD reflect a negative mood state, and high scores of vigor reflect a positive mood state (46).
  ○ VAS craving scale: a single-item 10-point Likert visual analog scale (from 0 to 9) assessing craving. The question is “How badly do you feel like smoking right now?”
  ○ Simulator Sickness Questionnaire: The 16-item SSQ was used to assess participants’ sickness levels before and after immersions in VR. Participants rated the severity of each symptom (e.g., dizziness, headache, sweating) on a 4-point Likert scale (0 – “None” to 3 – “Severe”) (47).
  ○ Presence Questionnaire: The PQ is a subjective perception of the environment that the individual experiences and is made of a total of 19 questions based on “involvement,” “immersion,” “visual fidelity,” and “interface quality.” (48)

For the control sample, all questionnaires will be administered in only one appointment.

### Statistical analysis

This protocol is a parallel group design.

Sample size:

The means for craving score, assumed to compute sample size, are reported in the following table.

**TABLE:** 

	Baseline	After 7 days	After 1 month
Control	4	None	none
Cytisine	6	4.5	4.5
Cytisine + Nyrdosh	6	3.5	3.5

Assuming the above-mentioned means and a 0.5 correlation between repeated measures, 26 tobacco-addicted individuals allow achieving 80% power to detect significant differences with a two-sided alpha of 5%.

### Statistical analyses

Significance of differences in craving score among the three groups will be evaluated by a repeated-measures ANOVA for mixed designs, where individuals will be the random effect, while the type of intervention and time (baseline/after 1 week/after 1 month) will be the fixed effects.

Estimated sample size for repeated-measures ANOVA and F test for between subjects:

Ho: delta = 0 versus Ha: delta! = 0.

Study parameters:

Alpha = 0.0500, power = 0.8000, and delta = 0.3600.

Estimated sample sizes: *N* = 78 N per group = 26.

Ethics Committee for Clinical Trials and approval code is 3624CESC.

## Discussion

Tobacco addiction is a public health problem, causing six million deaths per year (1). There are several different pharmacological treatments for TA currently available, but Cytisine seems especially promising due to it having several advantageous characteristics: it is low-cost, it has rare pharmacological interactions, it has an easy drug management, and a good efficacy and safety (49). The low cost of cytisine is also its “*Achilles’ heel*”: the long registration procedures in Western European (EU) countries and in the USA restrict Cytisine’s use to some Eastern EU countries. In most EU countries, and in Italy as well, there is the same paradoxical situation: the drug, albeit being registered in 4 EU countries (Poland, Bulgaria, Latvia and Lithuania) and 13 non-EU countries (Azerbaijan, Armenia, Belarus, Georgia, Kazakhstan, Kyrgyzstan, Moldava, Russia, Serbia, Tajikistan, Turkmenistan, Uzbekistan, and Ukraine), could be prescribed as a galenic formulation by physicians, but only a few of them know its proprieties and few pharmacists agree to formulating it (8). There are several studies about tobacco craving and VR, and they report that nicotine cues could increase cigarette craving (12). But tobacco addiction is not only a pharmacological problem: there are other aspects related to this addiction (such as sensory impact) which are crucial in comprehensively treating addiction and preventing relapses in smoking cessation. Using Nirdosh will allow us to understand if a cigarette tobacco substitute could be advantageous in tobacco detoxification treatments. Concerning possible results, we expect the C + N group to present less environmental craving levels than the C (Cytisine-only) group. Using Nirdosh in addition to Cytisine therapy may lower the sensitivity of this group to tobacco CR. In addition, we expect that the C + N group will present lower POMS questionnaire scores than the C group. We hypothesize that using Nirdosh will also lower the levels of TMD scores, pointing to a more positive mood state in this sample. Since data quality is very important, we will also administer two questionnaires that address possible cybersickness effects and evaluate simulation quality. Indeed, the quality of the simulation could have an impact on the sense of craving and the mood of the subjects, so it is important to take into consideration.

There are several limitations to this research: using Nirdosh makes it impossible to measure Co^2^ to observe tobacco abstinence, questionnaires are self-report measures and craving is measured using a self-report VAS scale.

## Conclusion

Tobacco addiction is a complex addiction. With this protocol we want to study non-pharmacological effects on craving and mood state in subjects who want to quit smoking. There are several studies that analyze tobacco craving, mood states and VR separately, but, to the best of our knowledge, there are no studies that examine the use of tobacco substitutes to manage tobacco craving during smoking cessation treatment by also using VR to measure craving and mood states.

## Author contributions

LZ, SCm, RG, MI, and FL were responsible for the study concept and design. RC, SM, FF, SCr, AC, ET, and IB drafted the manuscript. GV and RV were responsible for the study methodology. All authors critically reviewed the content and approved the final version of the manuscript for publication.
